# Impact of Intermittent Fasting on Metabolic Syndrome and Periodontal Disease—A Suggested Preventive Strategy to Reduce the Public Health Burden

**DOI:** 10.3390/ijerph192114536

**Published:** 2022-11-05

**Authors:** Sameena Parveen, Yaser Ali Alhazmi

**Affiliations:** Department of Maxillofacial Surgery and Diagnostic Sciences, College of Dentistry, Jazan University, Jazan 45142, Saudi Arabia

**Keywords:** intermittent fasting, metabolic syndrome, calorie restriction, periodontal diseases, inflammation

## Abstract

Metabolic syndrome (MetS) prevalence continues to climb significantly worldwide in today’s ad libitum society. MetS has tremendous societal and economic ramifications, making it imperative to develop effective strategies for preventing and controlling it to alleviate this growing burden. Periodontal disease and MetS are associated with several risk factors. Studies in the past have demonstrated that obesity, cardiovascular illness, and type 2 diabetes mellitus have a negative effect on the severity of the periodontal disease. Patients with metabolic syndrome have elevated serum levels of proinflammatory mediators such as tumor necrosis factor-alpha interleukin-6 and C-reactive protein. Similar inflammatory mediators, such as interleukin-6, tumor necrosis factor-alpha, and C-reactive protein, are increased in patients with severe periodontal disease. Remarkably, intermittent fasting is underpinned by scientific evidence, claiming to be the most effective non-pharmacological, potential therapeutic alternative for combating a wide range of metabolic, inflammatory, and lifestyle-related diseases. Nonetheless, an insufficient investigation has been performed to determine whether intermittent fasting has therapeutic benefits on periodontal inflammation and diseases. Here, we show the interrelationship between metabolic syndrome and periodontal disease and contextualize the beneficial impact of intermittent fasting in modulating the chronic metabolic and periodontal inflammatory response. We also anticipate that this review paves the way for further exploration of intermittent fasting as a unique research paradigm representing a cost-effective alternative strategy to conventional disease management in patients with periodontal diseases and metabolic syndrome which may serve as the foundation for an integrative vision relevant to primary, diagnostic, and therapeutic purposes.

## 1. Introduction

MetS is characterized as three of the five interconnected risk factors of diabetes and cardiovascular disease, namely high blood pressure (BP), elevated glucose levels, obesity (especially central adiposity), and low levels of high-density lipoprotein(HDL), or elevated triglyceride levels (TGS) [1]. Globally, the toll of all MetS components is skyrocketing because of increased incidence and prevalence [2]. One of the most important pathophysiological components of MetS is obesity, particularly central obesity, which is the root cause of all other metabolic abnormalities [3]. According to the Global Nutrition Report released in 2020, one out of every nine individuals worldwide suffers from hunger or malnutrition, and one out of every three is overweight or obese [4]. Patients with MetS have elevated serum levels of proinflammatory mediators such as tumor necrosis factor-alpha (TNF-α), interleukin-6 (IL-6), and C-reactive protein (CRP) [5]. Similar inflammatory mediators, such as IL-6, TNF- α, and CRP, are increased in patients with severe periodontal disease (PD). Glycemic management decreases the risk of PD in patients with type 2 diabetes [6], and CRP levels can be lowered when PD is successfully treated [7]. Previous studies confirm that obesity, CVD, and type 2 diabetes mellitus negatively influence PD severity and extent. Thus, cross-sectional findings demonstrated that sustaining a healthy body weight achieved through a well-balanced diet and regular physical activity dramatically reduces gingival inflammation and PD [8,9,10,11]. Several mechanisms have been proposed to relate MetS, obesity, and PD [8,12,13]. PD shares multiple genetic risk factors with CVS, in addition to being an independent risk factor [14,15,16]. MetS-associated inflammation has been linked to endothelial dysfunction, elevating the risk of cardiovascular disease and type 2 diabetes, although the underlying mechanisms driving this systemic response are not yet understood [13,17,18].

Intermittent fasting (IF) is an eating pattern that alternates between periods of fasting and periods of normal eating on a regular schedule [19,20,21]. IF incurs a net reduction in caloric intake, falling below the calories expended. It also leads to a negative energy balance that causes weight loss [22]. IF triggers neuroendocrine activation, adaptive cellular reactions, enhanced cellular repair mechanisms, increasing glycemic management, activation of the adenosine monophosphate protein kinase (AMPK) pathway, and sirtuins [20,23,24]. In addition, IF causes a reduction of mitochondrial oxidative stress, free-radical production, and signaling of insulin and mammalian target of rapamycin (mTOR), thus lowering inflammation [25]. Restriction of calories lessens the inflammatory response and decreases circulating proinflammatory mediators such as TNF-α, IL-6, matrix metalloproteinase-8 (MMP-8), matrix metalloproteinase-9 (MMP-9), and IL-1-beta in gingival crevicular fluid (GCF). To date, pre-clinical investigations have consistently shown that IF has a robust ability to alter chronic illnesses such as cancer, type 2 diabetes, overweight, heart ailments, and neurodegenerative brain disease [19,26,27,28,29].

Chronic metabolic illnesses have significant societal and economic repercussions, making it vital to find ways to avoid and regulate them. Since MetS and obesity are frequently associated with CVD and type 2 diabetes, there is an increasing need for exemplary low-cost, effective therapeutic solutions to alleviate this growing burden. The disease’s entire cost, including health treatment and reduced economic growth, surpasses trillions [2,4]. This motivates the need for an alternative novel approach that is practical and useful for resolving the problem. Modifications to one’s way of life and diet are at the forefront of the therapy of MetS. Remarkably, IF is underpinned by scientific evidence, claiming to be the most effective non-pharmacological, potential therapeutic alternative for preventing and treating chronic inflammation and disorders associated with a sedentary lifestyle [25]. Nonetheless, an insufficient investigation has been performed to determine whether intermittent fasting has therapeutic benefits on periodontal inflammation and disease. It is quite remarkable that this unique area of research has been largely untapped and neglected in the literature. A very negligible amount of information is available about the beneficial effects of fasting on periodontal health and disease at the moment. This clearly warrants the need for further exploration to know the systemic effects of IF on periodontal inflammation and components of MetS. We hope the present review provides a call for future research in developing IF as a unified approach, a new paradigm, a cost-effective adjunctive tool to conventional disease management in patients with both periodontal diseases and MetS.

Therefore, the present review aims to summarize the concept and understanding of the interrelationship between metabolic and PD and contextualize the beneficial impact of IF in modulating the chronic metabolic and periodontal inflammatory response. Additionally, we tried to emphasize the importance of critical elements such as mTOR, AMPK and SIRT (sirtuins) in the regulation of cellular and molecular pathways in the management of metabolic stress and energy intake.

## 2. Search Strategy and Selection Criteria

Search strategy and selection criteria through PubMed, Google scholar and Saudi Digital library for articles from inception until August 2021 using the following terms: “Intermittent fasting”, “Fasting”, “Alternate day fasting” or “ADF”, “Time restricted feeding” or “TRF”, “Periodic fasting”, “Ramadan fasting” in conjunction with “Periodontal diseases”, “Oral diseases”, “Periodontal inflammation”, “Chronic metabolic diseases” “Metabolic syndrome” or “Metabolic diseases”, “Diabetes” or “Diabetes Mellitus”, “Obesity”, “Obese”, “Weight” or “Overweight” “Calorie restriction “or “Low calories” to develop this review. Additional studies were identified by a manual search of bibliographic references in major papers and reviews and Google Scholar for additional references. Abstracts and non-English papers were not included.

## 3. Review Analysis

### Most Common Plans of Intermittent Fasting

**Figure 1:**
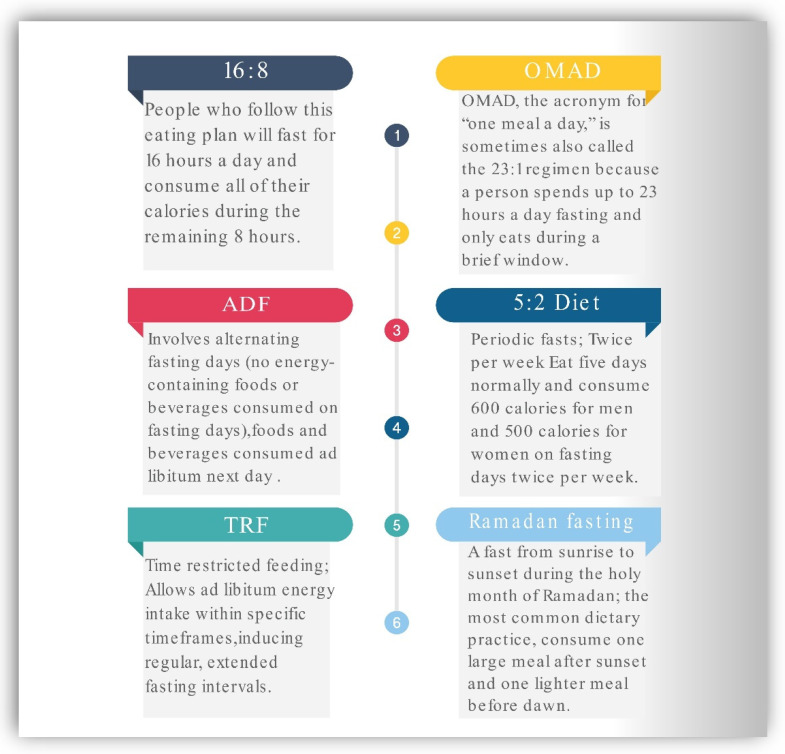
Most common Intermittent fasting regimens. ADF—Alternate-day fasting, TRF—Time restricted feeding, OMAD—one meal a day.

IF has been a popular health trend in recent years. The most prevalent approaches to this type of eating pattern are illustrated in Figure 1. The 16:8 strategy comprises fasting for approximately 16 h per day and eating during an 8 h window. The OMAD (One Meal a Day) is one of the extreme intermittent fasting methods. OMAD requires fasting for roughly 23 h a day and a short eating window of 1 h. Alternate-day fasting entails abstaining from food for approximately every other day, either by abstaining entirely or by ingesting only a few hundred calories. According to the 5:2 diet, one must eat regularly for five days out of the week while substantially reducing caloric consumption on the other two days of the week. Time-restricted feeding (TRF) is a dietary plan characterized by calorie consumption in a short interval limited to six to ten hours during the active period of the day without sacrificing food quality or quantity. During Ramadan fasting, it is customary to refrain from drinking or consuming any food between the hours of sunrise and dusk. In contrast, zero-calorie liquids such as water, tea, and coffee are often tolerated during other forms of intermittent fasting [21,30].

## 4. Cellular and Molecular Level Interactions during IF and Calorie Restriction (CR)

**Figure 2:**
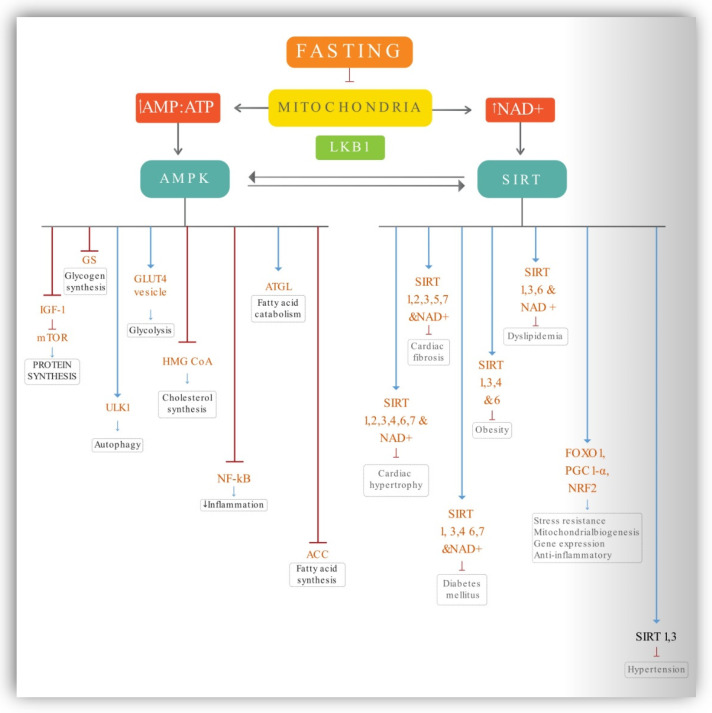
Molecular mechanism of action of fasting. Blue lines—activating action, Red lines—inhibiting action.

Several hypotheses have been proposed to explain the impact of prolonged fasting, particularly low-calorie diets, on a variety of molecular and cellular signaling pathways. These pathways and the ratio of bioenergetic sensors are activated and oscillated by the amount of energy consumed, the content of meals, and the length of fasting. This impact is mediated by higher activated protein kinase (AMP) and a decrease in cellular adenosine triphosphate (ATP), which results in AMPK activation, which inhibits various anabolic pathways and boosts catabolic processes. Exertion and energy deprivation activate AMPK, a critical regulator of energy metabolism [31]. Furthermore, nicotinamide adenine dinucleotide (NAD+) deacetylase activity of sirtuins (SIRTs) and AMPK deacetylation of LKB1 and SIRT1 trigger a feedback loop. A large number of downstream proteins that govern cell function and resistance to stress are activated by these intermediate energy carriers, such as forkhead box Os (FOXOs), peroxisome proliferator-activated receptor coactivator 1 (PGC-1), and nuclear factor erythroid 2–related factor 2 (NRF2). There are numerous metabolic health concerns that can be improved by the combination of these pathways. To better understand this process, a schematic representation of the most important primary energy sensor components that play a crucial part in molecular pathways signaling is depicted in Figure 2. Here, we summarize the potential action of key components that play a critical role during fasting and energy depletion.

Depletion of energy impairs mitochondrial function, resulting in a rise in the ratio of AMP to ATP and the concentrations of NAD+. The activation of AMPK is caused by an increase in the AMP: ATP ratio. LKB1 and SIRT1-mediated deacetylation of AMPK results in phosphorylation and activation of the enzyme, resulting in the establishment of a feedback mechanism. Reduced IGF-1 signaling and decreased circulating amino acids limit the activity of mTOR, resulting in protein synthesis suppression and autophagy promotion. AMPK activates ULK1, which activates the autophagy pathway. It also inhibits glycogen and fatty acid synthesis. AMPK HMG-CoA Reductase enzyme involved cholesterol synthesis. AMPK inhibits HMG-CoA. Activates glucose uptake through GLUT4 transporters, leading to increased glucose uptake and glycolysis. Increases fatty acid oxidation through ATGL. Furthermore, LKB1 and SIRT1-mediated acetylation phosphorylate/activates AMPK, resulting in a feedback loop. Cofactors for epigenetic modifiers like SIRTs include acetyl coenzyme A (CoA) and NAD+. SIRTs deacetylate FOXOs, NRF2, and PGC-1, causing stress resistance and mitochondrial biogenesis genes to be expressed. SIRT1, 3, 6, and NAD+ are involved in the regulation of dyslipidemia, while SIRT1, 3, 4, and 6 are involved in the prevention of obesity. SIRT1, 3, 4, 6, 7, and NAD+ may all have a role in type 2 diabetes protection. SIRT1 and 3 have been associated with decreased cardiac fibrosis, whilst SIRT2, 3, 5, 7, and NAD+ have been associated with decreased hypertension. SIRT1, 2, 3, 4, 6, 7, and NAD+ all contribute to the protection of the heart from hypertrophy.

### 4.1. AMPK Activation

The biochemical activation of AMPK occurs in the presence of increasing levels of AMP and decreasing levels of ATP, respectively. When AMPK is activated, cells do not make or store fat but rather burn fat for energy [32]. In response to energy levels, AMPK regulates both catabolic and anabolic processes and has a diverse array of targets. It also activates ATGL (adipose triglycerides lipase); fatty acid catabolism begins with releasing fatty acids from triglyceride molecules, which is the first enzyme implicated in that process. AMPK inhibits acetyl-CoA Carboxylases ACC1 and ACC2 through inhibitory phosphorylation, the enzyme responsible for production of fatty acid. As a consequence of phosphorylating and inhibiting ACC, AMPK depletes the malonyl-CoA pool; as a result, lipid synthesis is lowered, and fatty acid transport into the mitochondria for oxidation is enhanced [23]. AMPK inhibits HMG-CoA reductase enzyme (HMGCR) suppresses cholesterol synthesis by inhibiting enzymes involved in cholesterol synthesis. AMPK stimulates glucose transport via the GLUT4 transporter which inhibits glycogen storage. This leads to increase glucose uptake and glycolysis. There is a decrease in IGF-1 and mTOR activity during the fasting state. When there are sufficient nutrients, inactivation of AMPK and activation of mTOR occur. However, when nutrients are scarce, increased AMPK activity results in decreased mTOR activity. Reduced mTOR activation resulted in decreased cell growth and protein synthesis, implying that AMPK actually inhibits protein synthesis. AMPK stimulates the expression of the essential protein ULK1, which in turn initiates the autophagy process [33]. Increased glucose consumption, lipid mobilization, and macromolecule turnover through autophagy are among the mechanisms that have been activated. It induces mitochondrial biogenesis and stimulates the production of SIRT1, one of the sirtuins and an enzyme that repairs DNA. In response to decreased energy, SIRT1 is activated, which raises the cellular concentration of the NAD+ molecule [34]. AMPK inhibits the signaling of nuclear factor-κB (NF-κB), which is a proinflammatory pathway, so inhibiting this pathway results in reduced chronic inflammation [23,31].

### 4.2. Sirtuins

Sirtuins are a class of signaling proteins that function in metabolic regulation. Sirtuins are enzymes that help cells speed up chemical reactions. SIRT1 is regulated by NAD+ levels and is elevated under energy-deficient settings, resulting in NF-K downregulation and related transcription factors, which contribute to the reduction of inflammation [35]. At least in the case of the sirtuins SIRT1, SIRT3, SIRT4, and SIRT6, much research has been performed on the regulation of insulin and glycaemic control, as well as the onset and management of diabetes mellitus. SIRT1 depletion causes decreased liver X receptor expression and low levels of HDL and TG lipids, showing that SIRT1 plays a protective effect in increasing the amount of healthy HDL cholesterol in the body [24]. Fasting activates SIRT1 and SIRT3, which affect insulin response, antioxidant defense, and glycolysis. Due to their ability to change cellular metabolism, sirtuins may have anticancer effects that limit cell growth and make it resistant to the damaging effects of oxidative stress [36]. SIRT1 can aid in the prevention of the development of the Mets, obesity, and cardiomyopathy.SIRT3 has been shown to impact dyslipidemia and reperfusion damage positively [24]. Because sirtuins and NAD+ have a well-established role in cardiovascular and metabolic diseases, there is compelling experimental and clinical evidence that boosting SIRT1 activity can help treat type 2 diabetes and dyslipidemia. Strong scientific evidence supports SIRT6 activity in insulin resistance, SIRT3 activity in protecting against age-related cardiac hypertrophy, and NAD+ levels in angiogenesis and blood flow. These findings could pave the way for clinical studies in the future [37]. Sirtuins appear to have a complex but generally protective effect against obesity; increasing NAD+ levels or SIRT1 and SIRT6 activity may be a helpful therapeutic strategy for preventing and treating type 2 diabetes, MetS, and insulin resistance.

### 4.3. Nrf2, FOXO, PGC-1α

Nrf2 is involved in oxidative stress and toxicity; maintaining a suitable balance of reactive oxygen species (ROS) levels is critical for mitochondrial and all other pathways to function correctly [35]. Fasting-induced metabolic alterations in skeletal muscle result in a moderate increase in oxidative stress. The Nrf2 transcription factor is responsible for coordinating an adaptive program that helps maintain redox balance and increase glutathione synthesis [38]. As a result, nutrient restriction/fasting may be an effective technique for boosting antioxidant defenses and preventing lipid peroxidation.

FOXO1 is a conserved transcription factor regulating the overall body’s metabolic activity [39]. The importance of FoxO1 in energy balancing becomes even more apparent when there is a metabolic disorder or insulin resistance. Several of the negative features linked with obesity and diabetes, such as hyperglycaemia and glucose intolerance, are promoted by FoxO1-dependent gene expression [40]. Metabolic dysfunction associated with fatty liver disease and atherosclerosis is just some of the metabolic conditions that can lead to diabetes, obesity, or both, which are caused by FoxO1 pathway dysfunction [39].

PGC-1α is an important transcriptional coactivator for mitochondrial biogenesis and function, detoxification of ROS, and oxidative phosphorylation. This transcriptional coactivator, which has been named “the master regulator of mitochondrial biogenesis,” regulates the production and function of mitochondria. PGC-1 expression, which is prevalent in tissues with high energy needs, is clearly associated with the pathogenesis of the Mets and its major consequences, such as CVS, obesity, and type 2 diabetes [41].

## 5. Impact of IF on PD and Mets

### 5.1. Periodontal Diseases

**Figure 3:**
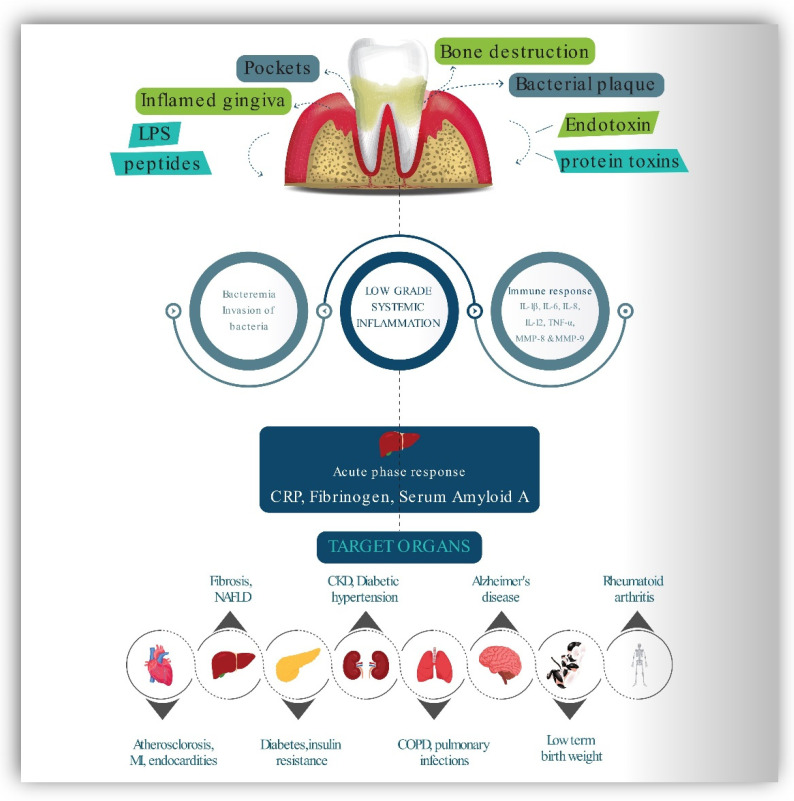
The relationship between periodontal disease and other systemic disorders.

PD is a chronic inflammatory condition that causes inflammation of the periodontal soft tissues and progressive loss of periodontal ligament and alveolar bone, destroying the tooth-supporting apparatus and potentially resulting in tooth loss [42]. Inflammation is the underlying cause of periodontal diseases, and it plays a critical role in their progression. The presence of a considerable number of bacteria might indirectly result in tissue degeneration by activating host defense cells, which produce and release chemical mediators that promote connective tissue breakdown effectors. Microbial plaque components have the ability to cause an initial infiltrate of inflammatory cells such as lymphocytes, macrophages, and polymorphonuclear leukocytes (PMNs). Microbial components, particularly lipopolysaccharide (LPS), stimulate macrophages to produce and secrete a wide range of proinflammatory molecules, including the cytokines, interleukin-1 IL-1, IL-4, IL-10, IFN, TGF and TNF-alpha; prostaglandins, particularly prostaglandin E2 (PGE2); and hydrolytic enzymes. These cytokines have strong proinflammatory and catabolic properties, and they play an important role in the degradation of periodontal tissue through the action of collagenolytic enzymes such as MMPs [43]. ROS in the inflammatory environment activates these latent collagenolytic enzymes [44]. While it is possible that inflammatory processes originating in one organ induce disorders in another, communication between distant sections of the body and their inflammatory states are accomplished by cells or soluble chemical mediators [42]. Significant evidence has been gathered to support comorbidity between PD and other chronic illnesses such as diabetes mellitus, CVS, preterm birth, rheumatoid arthritis, respiratory diseases, chronic kidney diseases, and Alzheimer’s. Figure 3 demonstrates proposed connections between PD and several systemic diseases using schematic representation [13,42,45,46,47,48].

The periodontium responds to the dental plaque by the release of bacterial lipopolysaccharides (endotoxins), chemotactic peptides, protein toxins. These molecules stimulate the host to produce a variety of responses with the production of inflammatory mediators IL-6 IL-1β, IL-6, IL-8, IL-12, TNF-α, MMP-8 & 9, PGE2 and fibrinogen. This type of bacteremia causes the liver to initiate an inflammatory and immunological response, resulting in higher blood levels of c-reactive protein and the production of serum amyloid A and fibrinogen. These and other host products response may influence a variety of essential disease pathways and are capable of initiation and development of chronic systemic diseases in target organs. COPD—Chronic obstructive pulmonary disease, NAFLD—Non-alcoholic fatty liver disease, MI—Myocardial infarction, PGE2- Prostaglandins, MMP—Matrix metalloproteinase, IL—Interleukin, TNF-α—tumor necrosis factor-alpha, CRP—C-reactive protein.

### 5.2. Activation and Regulation of Periodontal Inflammation by AMPK Pathway and Role of Sirtuins

Oxidative stress is a critical regulator of the systemic pathophysiological effects of PD. SIRT1’s anti-oxidative stress effects in PD have been elucidated. The activation of SIRT1 phosphorylates and activates AMPK, thereby reducing periodontal inflammation-induced oxidative stress. Significantly decreased alveolar tissue damage, enhanced cell viability, and decreased release of proinflammatory cytokines such as IL-1, IL-6, and TNF were found. SIRT 2 was also found to be positively related with nicotinamide phosphorribosyl transferase (NAMPT) activities in human gingival fibroblasts, which were greatly elevated during PD and involved in osteoclast recruitment by promoting the production of cyclooxygenase-2, matrix metalloproteinase (MMP)-1 and MMP-3 [49]. In a study involving mice, Chen et al. show that SIRT3 inhibits oxidative stress through controlling PGC-1 and mitochondrial function [50]. SIRT6 is also implicated in the regulation of periodontium homeostasis in PD. Huang et al. demonstrated that elevated SIRT6 inhibits cementoblast development and mineralization via inhibiting glucose transporter 1 (GLUT1), a glucose transporter required for cementogenesis, and by activating the AMPK pathway. SIRT6 overexpression improves osteogenic differentiation and reduces LPS-induced inflammatory response through inhibiting the NF-B pathway [51].

We predicted that fasting may halt the course of PD and lessen systemic oxidative stress through the activation of mitochondrial sirtuins and the AMPK pathway. By reducing oxidative stress, fasting may help prevent alveolar bone loss and inflammatory reactions in the periodontium. The exact roles played by IF at the molecular level to inhibit initiation and progression of PD still require further investigation. However, defining the function and mechanism of fasting in the context of PD may be very helpful for the development of novel therapeutic approaches.

### 5.3. Impact of Intermittent Fasting and Calorie Restriction on Periodontal Inflammation Diseases

Numerous dietary suggestions that improve periodontal health are vitamin C and vitamin D3 dietary supplements, omega-3 complex supplementation, fiber supplements, antioxidant supplementation, and carbohydrate restriction [52]. Regardless of whether persistent plaque is present, anti-inflammatory regimes have been shown to considerably reduce inflammation of gingival tissue [11]. Recent cross-sectional research reveals that healthy body weight, nutritious food, and adequate physical exercise reduce the severity and extent of PD [53]. However, in humans, the effect of IF and calorie restriction on periodontal inflammation and infection is scarce. The long-term, irregular, and unpredictable nature of the periodontal disease progression makes disease evaluations extremely difficult. The inflammatory, immunological, kinetic, and disease progression characteristics of active periodontal destruction were studied in different animal studies [54].

A recent study [55] used an experimental mouse model to assess the positive benefits of IF regimens for periodontal tissues. The authors discovered that fasting regimens resulted in a decreased loss of bone than non-fasting regimens at the ligature-induced PD site and on the contralateral maxillary side. Quantitative computed tomography and calcein-labeled histomorphometric examinations on peripheral bone revealed that the fasted sample subjects had a more excellent capability for regenerating bone than the non-fasted sample group. Additionally, the bone marrow cells of the fasted groups formed more incredible mineralized modules than those of the non-fasted groups [55].

Nonhuman primates are ideal animal models for periodontics research owing to their anatomical and biological similarities. The National Institute on Aging on a Rhesus monkey cohort (Macaca mulatta) conducted a longitudinal to ascertain the influence of calorie restriction on the clinical microbiological and immunological aspects of PD. Monkeys were fed twice daily at 6:30 a.m. and 1 p.m. (16 h fasting period and 8 h eating window, 16:8 ratio). For 13–17 years, monkeys were subjected to a 30% drop in dietary consumption of calories compared with a control ad libitum diet group [56,57,58]. The results proved that long-term exposure to CR and time-restricted eating significantly reduced the degree of naturally occurring chronic periodontitis. The CR diet reduced the ligature-induced gingival index (GI), probing pocket depth (PPD), bleeding on probing (BOP), and clinical attachment level (CAL) by a significant amount [56].

Another study [58] found that the male CR group had significantly decreased depth of the periodontal pocket, substantially lower levels of IgG antibody response, and considerably decreased IL-8 and -glucuronidase levels in GCF in comparison to a control group that received ad libitum meals. However, the male CR group showed a non-significant drop in the IL-1 levels. In the GCF, the female calorie-restricted group had reduced IgG levels of antibodies than the ad libitum group, but both groups had equal levels of markers of inflammation, suggesting that calorie restriction may be beneficial. However, the periodontal microbiota of male and female monkeys was unaffected by a calorie restriction diet [58]. In another study conducted to know the antibody responses and also the acute serum host response in rhesus monkeys, it was demonstrated that gender variations in calorie restrictions have an impact on systemic effects. Samples of male monkey serum had higher quantities of haptoglobin and a1-acid glycoprotein than the sample of female monkey serum, according to the findings. Serum IgG antibody responses to *Pophyromonas gingivalis*, *Campylobacter rectus*, and *Actinomycetemcomitans* were significantly increased in the sample of female rhesus monkeys. Antibodies against *Fusobacterium nucleatum*, however, demonstrated a substantial impact in females on a calorie-restricted regime [57]. Recent findings from the first human trial indicated that a specified therapeutically supervised periodic fasting regimen had beneficial effects on inflammation of periodontal tissues in female MetS patients.

Bodyweight, waist circumference (WS), body mass index (BMI), BP, fasting plasma glucose (FPG), total cholesterol, CRP, and HDL were all reduced as a result of clinically supervised fasting. At the same time, BOP and GCF levels were also reduced as a result of the fasting [59]. As a result, fasting may be recommended as an additional strategy in addition to routine periodontal therapy in obese and overweight individuals who have been diagnosed with periodontitis. Therefore, it will be imperative to conduct many randomized clinical trials in persons who have MetS and periodontal disease in the future. Figure 3 presents a schematic representation of the impact of IF on CVS, metabolic, and the periodontal health

### 5.4. Evidence of Correlation between MetS and PD

The published literature was combed for case–control, cross-sectional, and cohort studies involving individuals with MetS and PD measures. We looked for studies published before December 2020 in PubMed, Web of Science, Science Direct, and Google Scholar that assessed the relationship between PD and MetS. Numerous studies found linkages between the severity and incidence of PD and obesity [60,61]. In addition, type 2 diabetes is a risk factor for PD [62,63]. Prevalence, severity, and risk of PD are all connected to high blood sugar levels [64]. Glycemic control reduces the PD risks in people with diabetes [6], revealing that the connection between diabetes and PD is bidirectional [7].

Some studies also linked PD to obesity, dyslipidemia, hypertension, and hyperglycemia (all of which are components of MetS) [16,53,65,66,67,68]. Another analysis found a strong correlation between gingival inflammation and low HDL cholesterol in the teens who meet the MetS criteria [69]. Another study [70] discovered a link between periodontal disease and HDL cholesterol levels in female adults. As a result, PD is identified as an important component of MetS, frequently altered in diabetes and other systemic disorders [71]. The overwhelming volume of data suggests that PD Mets are linked. Table 1 summarizes the studies reviewed, highlighting the correlation between PD and MetS.

### 5.5. Inflammation and Immune Mediation between PD and MetS

Inflammation contributes a significant part in the initiation of the MetS [106]. Numerous studies established correlations between inflammation and obesity. Increased levels of CRP, TNF, IL-6, fibrinogen, and other acute-phase reactants have been documented in obese people [107,108,109,110,111,112,113]. Several diseases, such as hyperlipidemia [114], PD [115,116], and type 2 diabetes mellitus [117], are correlated with elevated cytokine production. Increased IL-1 levels in GCF [118] were observed in patients with insulin depended diabetes and hyperlipidemia, thus, a vicious cycle may develop. Inflammation caused by PD and MetS may further aggravate and exacerbate PD and metabolic sickness [119,120,121,122], resulting in impaired metabolic regulation and type 2 diabetes-related problems. [119] Furthermore, TNF, a cytokine that causes insulin resistance [123,124,125,126,127,128], is dose-dependent and linked with the severity of PD in adult individuals with insulin-dependent diabetes [129]. Thus, the hyper inflammatory state syndrome can amplify the local and systemic inflammatory responses to microbes. The response of the host to the microbial assaults caused by cytokine dysregulation is associated with prolonged TNF expression [130,131].

According to multiple animal and human studies, diabetes may accelerate alveolar bone disintegration via hyperglycemia-mediated modulation of the receptor activator of nuclear factor-kappa B ligand to osteoprotegerin ratio in periodontal tissues [132,133,134]. The rate of osseous regeneration following bone resorption may be reduced as a result of the death of bone-lining cells and the increased number of fibroblasts [135,136,137]. As a result, all of these factors may have a role in the uncoupling of bone breakdown and healing in periodontal diseases, which are commonly noticed in patients with type 2 diabetes. Increased blood glucose levels result in the formation of advanced glycation end products, which bind to the periodontal receptor for advanced glycation end products (RAGE) and initiate an inflammatory response [138]. Blocking the RAGE receptor in diabetic mice reduced the inflammatory response and the subsequent loss of alveolar bone [139]. Insulin resistance in PD patients may be associated with the host’s inflammatory response to lipid alterations, obesity, and periodontal diseases [140]. Reduced amounts of leptin, an anti-obesity adipocytokine in the gingiva, and GCF can aggravate PD [141,142,143]. However, serum leptin levels, on the other hand, tend to rise as periodontal disease progresses [143]. Despite the fact that an in vitro study revealed that adiponectin might have an anti-osteoclastic effect on PD [144]^,^ evidence for its anti-inflammatory effect between PD and MetS is limited and conflicting [145,146,147]. Resistin is an adipocytokine that is associated with proinflammatory properties [148] and is linked to insulin resistance [149]. Additionally, when individuals with PD were compared with people who were healthy, there was a link between bleeding on probing and higher levels of serum resistin [146,147].

### 5.6. Impact of Intermittent Fasting on MetS

As previously stated, short- and long-term IF greatly reduces inflammatory mediator CRP and cytokines such as IL-6 [150]. As a result, it is hypothesized that fasting improves the characteristics of MetS via modulating inflammatory responses. Ramadan fasting can decrease TNF- and IL-6 expression in healthy volunteers as it leads to weight loss and decreases body fat percentage [151]. In addition, it is hypothesized that TNF- and IL-6 limit lipoprotein lipase (LPL) action, resulting in TNF- and IL-6 down-regulation in fasting individuals, increasing LPL activity and decreasing the fat mass of the body [152].

A substantial body of scientific evidence using animal models suggests that IF enhances insulin sensitivity. When fasting or exercising for an extended period of time, the hepatic, cardiac, and skeletal system shift their metabolism to fatty acid oxidation and catabolism of amino acids. However, an energy-dense condition, on the other hand, favors glucose absorption and oxidation [153]. IF induces the gene expression that involves lipid storage (PPAR 2 and Fsp27) and fat oxidation (MCPT1), thereby increasing lipogenesis during the IF unrestricted phase and boosting metabolic flexibility and fat oxidation during the fasting period [154].

Insulin is critical for glucose homeostasis because it promotes glucose storage. Several explanations have been proposed to elucidate how insulin resistance develops. Prevalent hypotheses suggest that obesity is associated with chronic inflammation, leading to insulin resistance in tissues [155]. Growing evidence suggests that obesity is associated with chronic inflammation, which leads to insulin resistance in tissues [154]. So, through CR and metabolic reprogramming, IF helps to lower obesity and insulin resistance. Furthermore, various studies have proven that IF results in decreasing levels of leptin and increasing adiponectin, thereby improving insulin resistance [156].

**Figure 4:**
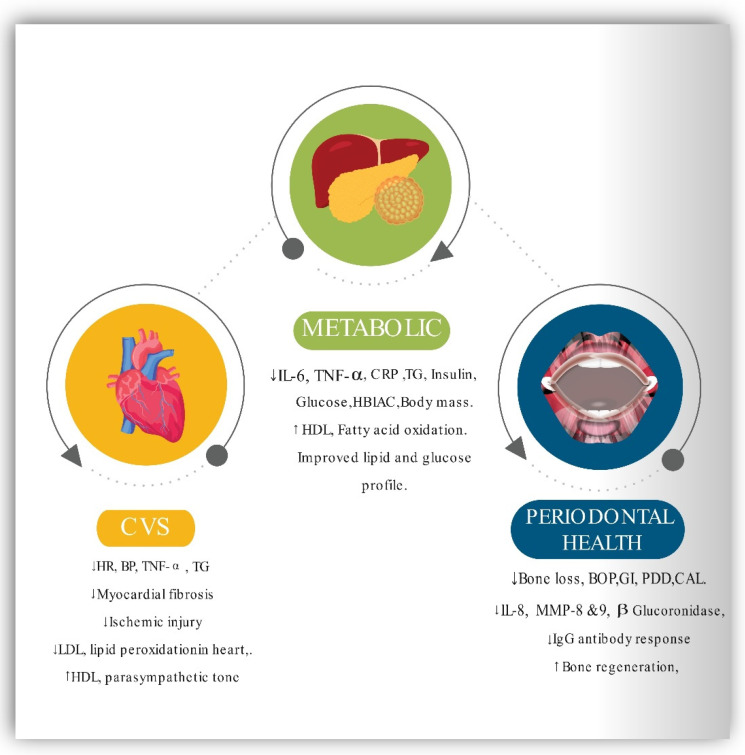
Schematic representation of the effect of intermittent fasting on CVS, Metabolic and Periodontal health. CRP—C-reactive protein, HDL—High-density lipoprotein, LDL—low-density lipoprotein, IL-6 Interleukin-6, Hb1Ac—Hemoglobin A1c, BOP—bleeding on probing, PPD—probing pocket depth, GI—Gingival index, CAL—clinical attachment loss, IgG—Immunoglobulin, BW—body weight, BMI—body mass index, WC—waist circumference, FGLU—fasting glucose, TRG—triglycerides.

Therefore, CR helps individuals to lose weight and improve metabolic health [157]. Numerous studies have revealed that humans face difficulties in maintaining daily CR for prolonged periods [158]. However, IF has a higher compliance rate and is effective in weight loss and the reduction of obesity-related risk factors for metabolic disease [21,159,160]. Because of the positive effects of fasting, the body uses fatty acids and ketones as fuel. As shown by research, altering one’s metabolic process to one that utilizes fatty acids for energy rather than glucose preserves muscle mass and function while increasing one’s ability to perform daily activities [159]. Finally, IF has been shown to reduce adipose tissue mass, notably visceral and abdominal fat, due to its mild energy deficits [161,162]. This effect has the additional effect of improving the cardiovascular risk profile by lowering BMI and blood pressure, decreasing resting heart rate, decreasing ischemic injury, decreasing lipid peroxidation, and enhancing cardiovascular stress adaptation and resistance to a cardiac muscle injury in myocardial infarction animal models. CVS risk factors are intimately related to MetS components, and intermittent fasting may play a crucial role in the prevention and management of CVD and MetS [25,159,162]. Figure 4 represents the beneficial effects of IF on CVS, metabolic and periodontal health.

## 6. Contribution, Significance, and Implications of the Current Review

**Figure 5:**
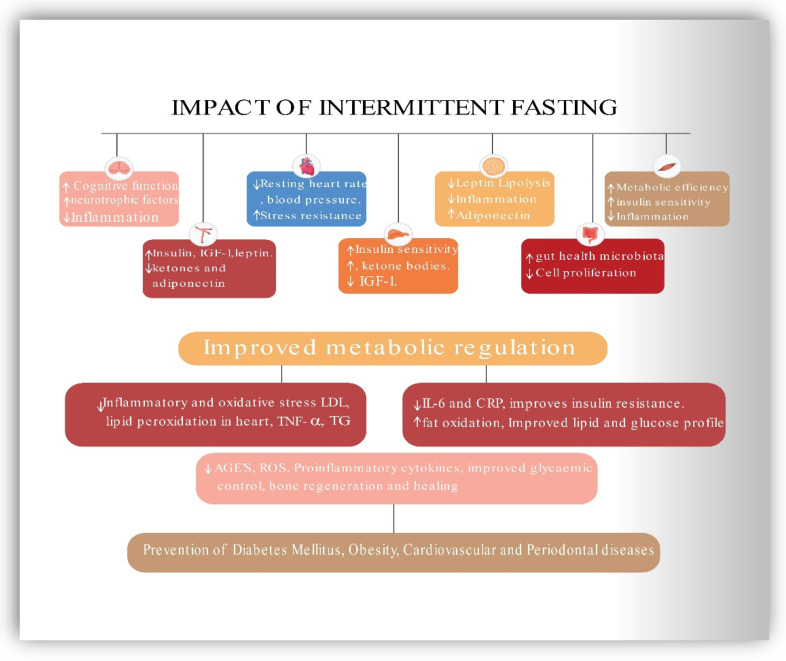
Schematic representation of the beneficial role of Intermittent fasting in preventing chronic diseases. IGF-1—Insulin-like growth factor, AGE’S—Advanced glycation end product, ROS—Reactive oxygen species, IL-6 Interleukin-6, CRP—C-reactive protein, LDL—low-density lipoprotein, TG—triglycerides.

This review signifies IF as a therapeutic intervention capable of addressing numerous behavioral risk factors in individuals and the population with favorable outcomes rather than addressing a single risk factor associated with a specific disease. The findings advance our understanding of the relationship between metabolic and periodontal conditions, as well as the therapeutic effects of IF concerning the metabolic and periodontal inflammatory responses. We hope that this study paves the way for further exploration of IF as a unique research paradigm representing a cost-effective alternative strategy for shifting the population’s health profile in a more favorable direction. Herein, we also conceptualize in a diagrammatic presentation based on studies of laboratory animals and human subjects the robust disease-modifying efficacy of IF on various organs of the body, including the brain, gut health, muscle, and blood vessels, adipose tissue, liver, and heart [19,30,106,159]. Figure 5 represents a schematic representation of the impact of IF in preventing chronic metabolic and inflammatory diseases like PD, obesity, CVS, and type 2 diabetes.

## 7. Current Research Gaps and Limitations

It should be noted that this is not a systematic study and, as a result, does not have the potential to summarize all research trials that are statistically significant. Having stated that, we wanted to draw attention to the fact that despite evidence that IF improves cardiovascular health and insulin sensitivity by combining weight reduction and “metabolic reprogramming,” there seems to be little research on the influence of low-calorie diets and IF on oral health, with the majority of the data coming from animal studies. Finally, it is critical to examine individuals for whom fasting is not recommended. These include pregnant/lactating women, elderly adults, those with immunodeficiencies, people who have hypoglycemia episodes, and people suffering from eating disorders.

## 8. Future Directions

IF is a future horizon that must be explored as a primary preventative tool in managing MetS and lifestyle-related disorders. These regimens may have the greatest impact on public health when implemented in large populations to counteract the growing deleterious impacts of obesity, fatty liver disease, MetS, and prediabetes. While underlying science and some clinical evidence of remarkable success support IF, the studies have been small and short-lived. More research on IF regimens in people with MetS and prediabetes PD is anticipated. To achieve long-term success, governments must collaborate with the commercial sector, health professional organizations, consumer groups, academia, the research community, and other non-profit organizations.

## 9. Recommendations for Intermittent Fasting in Practice

**Figure 6:**
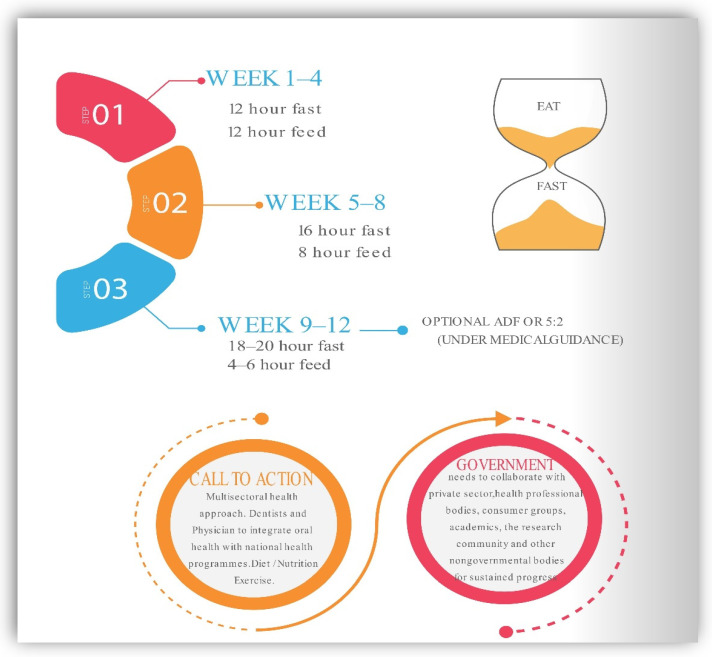
Suggested integrated approach to practice IF as a preventive interventional strategy.

We suggest widespread adoption of IF can be vital in the medical, dental, and nutrition communities to improve human health, particularly in connection with excessive eating habits and a sedentary lifestyle. Facilities that support patients in the transition to sustainable IF programs and routines are crucial, which include food, nutrition, exercises, and psychological assistance. It is essential to understand the social determinants like ever flashing commercial advertisements of fast food, dietary globalization, and decreased general physical activity as current barriers. Changing present trends will necessitate a diversified approach. Additionally, individuals who begin an intermittent fasting diet may feel initial discomfort, hunger, and irritability; however, these symptoms often decrease after a few weeks [25]. As a result, it is preferable to begin gradually. For instance, physicians may counsel patients to restrict their consumption to 12 h each day. Once patients become accustomed to this eating pattern, the feeding window may be reduced to 8 h as this provides the patient with daily calorie flexibility, which improves compliance. After becoming accustomed to eating on a scheduled plan of IF, patients may move to fast on an alternate day or periodic fasting under the guidance of a professional nutritionist. A multisectoral approach is needed to halt the escalation of MetS epidemics. An ounce of prevention is worth a pound of cure. Fasting procedures that are well-structured and tailored can help lower the risk of metabolic disorders. Figure 6 presents the suggested integrated approach to adapting IF as a preventive interventional strategy.

## 10. Conclusions

Prevention is key for transforming both the oral and overall healthcare systems. Obesity, frequent eating (ad libitum), and physical inactivity are substantial risk factors for MetS. Many studies have identified a connection between the severity of PD and the MetS. The current global anticipated expenses for MetS associated with frequent eating and physical inactivity far outweigh all other health costs. For individuals who can fast for a few hours or days a week, IF programs may be a potentially cost-effective, alternative, and desirable approach to improve cardio-metabolic health and oral health. Encouraging fasting to address numerous behavioral risk factors may be more beneficial to individuals and the general public than addressing a single risk factor associated with a single disease over a prolonged timeframe.

## Figures and Tables

**Table 1:** Correlation between PD and MetS.

Periodontal Disease Parameters	Metabolic Disease Parameters	Study Design	Author & Year	The Outcome
PD, CAL, ABL, tooth Mobility	BP, TG, FPG, HDL, and WC.	Longitudinal study Study duration: 33 years sample size: 760	Kaye et al., 2016 [72]	PD may be exacerbated or developed as a result of MetS.
PD and ABL	HDL, BP, WC, FPG, and TG,	Longitudinal study Study duration: 15 years Sample size: 1964	Tegelberg et al., 2019 [73]	PD was linked to MetS in an exposure-dependent manner.
PD (CPI)	BMI, BP, TG, HDL, TC, and FPG.	Longitudinal study Study duration: 4 years Sample size: 1964	Morita et al., 2010 [74]	PD was linked to greater conversion of MetS components.
CPI	BP, FPG, TG, HDL, and WC.	Longitudinal study Study duration: 2 years Sample size: 390	Sakurai et al., 2019 [75]	Positive MetS components were more prevalent in those with progressive PD than in those without/improved PD.
BOP, PD, Plaque, Recession	CRP, FPG, TG, TC, LDL, Pregnancy, weight, BMI, BP, HbA1c, and HDL	Longitudinal study Study duration: 3 years Sample size: 188	Bullon et al., 2009 [76]	PD and MetS are linked.
CPI	HDL, BP, WC, FPG, and TG.	Longitudinal study Study duration: 1 year Sample size: 136	Adachi et al., 2020 [77]	The development of the MetS did not appear to be connected to periodontitis.
CAL, BOP, PD	TG, WC, FPG, HDL, and BP.	Longitudinal study Study duration: 8/16 year Sample size: 539	Nascimento et al., 2019 [78]	MetS and PD showed a favourable link when latent variables were used to account for the many aspects of each disease. In terms of observable characteristics, MetS and PD were not linked.
CPI	HDL, and FPG Abd obesity, BP, and TG,	Longitudinal study Study duration: 3 years. Sample size:125	Iwasaki et al., 2015 [79]	The MetS have been linked to a higher risk of PD in older Japanese adults.
PD, CAL, BOP, and PI	HDL, Abd obesity, FPG/or T2DM, TG, and BP	Case-control No of patients: 208 Age of the patients: 37 to 78	Li et al., 2009 [80]	PD was known to be correlated with MetS even when other risk factors were treated in patients with the condition.
CPI	FPG, dyslipidemia, BP, and BMI	Cross-sectional No pf patients: 1315 Age of the patients: 30 to 92	Borges et al., 2007 [81]	PD patients had a higher prevalence of MetS, although the difference was not statistically significant.
CAL, GI, PD, and PI	HDL, TG TC, BP, FPG, and WC	Case-control No of patients:156 Age of the patients: ≥25 or above	Khader et al., 2008 [82]	Compared to patients without MetS, patients with MetS had more frequent and severe periodontitis.
CAL and PD.	TG, BP, FPG, Abd obesity, and HDL	Case-control No of patients: 584 Age of the patients:40 to 79	Shimazaki et al., 2007 [70]	MetS increases the risk of PD.
PD and BOP	HDL. BP, TG, WC, and insulin resistance	Cross-sectional No of patients: 13,677 Age of the patients: ≥17	D’Aiuto et al., 2008 [83]	Severe PD has been connected to MetS in adults in their mid-twenties.
PD.	TG, HDL, FPG/or Med, Abd obesity and B.P./or Med.	Cross-sectional No of patients: 7431 Age of the patients:20 to 90	Andriankaja et al., 2007 [84]	In females, this research revealed a substantial correlation between MetS and periodontitis. It was found that both sexes were affected by abdominal obesity as a metabolic factor.
CPI	TG, WC, BP, HDL. TC, FPG, BMI, and HbA1c.	Cross-sectional No of patients: 2478 Age of the patients: 24 to 60	Morita et al., 2010 [74]	In Japanese employees aged 20 to 60, there was a link between periodontal disease and MetS.
CPI	BP, HDL, FPG, TG, and obesity	Cross-sectional No of patients: 1070 Age of the patients: 40 to 70	Kushiyama et al., 2009 [85]	The more MetS components, the worse the situation. and the greater the risk of developing severe periodontitis.
CAL, PD, PI, and GI	BP, HDL, WC, TG, and FPG	Cross-sectional No of patients: 276 Age of the patients:35 to 74	Benguigui et al., 2010 [86]	Diabetes and PD are linked, with insulin resistance playing a significant role.
PD	BP, Insulin resistance, dyslipidemia and Abd obesity	Cross-sectional No of patients: 20 & 50 Age of the patients: 30 to 64	Timonen et al., 2010 [87]	Numerous components of the MetS were shown to be weakly linked to periodontal disease and dental caries in this research.
PD, BOP, and calculus	FPG, BP, Abd obesity, TG, and HDL	Cross-sectional No of patients: 1046 Age of the patients: ≥18	Han et al., 2010 [88]	PD and MetS may be linked. Age, gender, and smoking all played a significant role. The MetS with elevated glucose and hypertension had a more substantial impact.
BOP, CAL, and PD	FNG, HDL, T.G., W.C., and B.P.	Cross-sectional No of patients: 2370 Age of the patients: 40 to 79	Furuta et al., 2013 [89]	There appear to be gender disparities in PD and MetS. As a result of MetS, women may be more susceptible to developing PD compared to men
GI, PI, and PDI	WC, BP, TG, HDL, FPG, or T2DM and TC.	Cross-sectional No of patients: 253 Age of the patients: >18	Chen et al., 2011 [90]	In haemodialysis patients, moderate-to-severe PD is linked to MetS.
ABL	TG, FPG, WC, and BP.	Cross-sectional No of patients: 190 Age of the patients: mean: 56.8 ± 12	Nesbitt et al., 2010 [91]	Individuals with symptomatic PD had a 2.5 times higher chance of developing MetS.
CAL, and PD	BP, FPG, TG, and HDL and obesity	Cross-sectional No of patients: 6421 Age of the patients: 34 to 77	Fukui et al., 2012 [92]	Periodontal health is linked to MetS, especially in people suspected of having an untreated, periodontal disease.
PD	BP, HDL, WC, FPG, and TG.	Cross-sectional No of patients: 7178 Age of the patients: ≥19	Kwon et al., 2011 [93]	PD had a 1.55 odds ratio of being related to MetS.
PD, CAL, and ABL	TG, FPG or Med, HDL, Abd obesity, BP or Med,	Cross-sectional No of patients: 657 Age of the patients: 50 to 79	LaMonte et al., 2014 [94]	In this group of postmenopausal women, there was no consistent relationship in terms of MetS and periodontitis.
CAL and PD.	BP, dyslipidemia, BP and WC	Cross-sectional No of patients: 234 Age of the patients: ≥80	Minagawa et al., 2015 [95]	The researchers discovered a link between PD and MetS.
CAL, BOP, and PD	BP, HDL, WC, FPG, and TG.	Cross-sectional No of patients: 419 Age of the patients: 24 to 89	Gomes-Filho et al., 2016 [96]	The findings of this study suggest that severe PD is associated with MetS and vice versa.
BOP GI, PI, PD, and CAL	BP Glucose tolerance TG, HDL, and BMI	Case-control No of patients: 651 Age of the patients:	Jaramillo et al., 2017 [97]	PD and MetS have a positive relationship. The adjusted odds ratio is 2.72. Glucose sensitivity is a strongly related factor.
CAL and PD.	TG, HDL, BMI, WC, BP, and FPG	Cross-sectional No of patients: 5078 Age of the patients: 50 to 94	Kim et al., 2018 [98]	The MetS was shown to be more common among Korean people whose PD had worsened.
CAL, PI, BOP, and PD	TGs LDL, BP and/ or WC	Case-control No of patients: Case: 122 Controls: 366 Age of the patients:	Campos et al., 2020 [99]	There is a greater prevalence, severity, and development of PD among persons with MetS.
BOP, Plaque, PD, and CAL	BP, FPG (OGTT), HDL, TG, and Abd obesity	Cross-sectional No of patients: 283 Age of the patients: 26 to 87	Sora et al., 2013 [100]	The MetS are associated with the severity of PD in this Gullah group of people with type 2 diabetes.
CAL, BOP, and PD	HDL, BP, FPG, WC, and TG,	Cross-sectional No of patients: 125 Age of the patients: 35 to 76	Thanakun et al., 2014 [101]	Severe PD was connected to MetS in this Thai population.
CPI	FPG, HDL, Abd obesity, BP, and TG.	Cross-sectional No of patients: 125 Age of the patients: 35 to 76	Chen et al., 2010 [88]	MetS was prevalent enough to be deemed a medical disorder, and it was associated with PD.
BOP. PI, GI, PD, and CAL	TG, BP HDL, FPG and WC	Cross sectional No of patients: 363 Age of the patients: 18 to 81	Musskopf et al., 2017 [102]	PD and MetS have a weak relationship. The correlation is seen in people between the ages of 41 and 60.
CPI	HDL, TG and Med, FPG/and BP and Med	Cross-sectional No of patients: 1856 Age of the patients: mean: 66.4	Kikui et al., 2017 [103]	PD is linked to MetS and low HDL cholesterol. PD was found to be more common in people who had two or more MetS components.
BOP, CAL, PI, GI, and PD	FPG, BMI, WC, HDL, and BP.	Cross-sectional No of patients: 412 Age of the patients: mean: 57.8 ± 5.7	Pham et al., 2018 [104]	The severity and extent of PD raised with the number of MetS components. Periodontal variables were connected to increased MetS risk.
CPI	HDL TG, Obesity, BP and FPG	Cross-sectional No of patients: 1070 Age of the patients: 40 to 70	Kushiyama et al., 2019 [85]	The more components of the MetS present, the greater the chance of developing severe PD.
CAL of ≥ 3mm and ≥4 teeth with ≥ 4mm	HDL, Abd obesity, TG, FPG and BP.	Longitudinal study Duration: 1 year No of patients: 165 Age of the patients: 35 to 65	Lopez et al., 2012 [105]	MetS patients who underwent root planning, systemic antibiotics, plaque removal, and subgingival scaling after nine months had lower CRP levels.

## Abbreviations

AMP: Adenosine monophosphate, AMPK: Activated protein kinase, ACC: Acetyl-CoA Carboxylases, NAD: Nicotinamide adenine dinucleotide, ATGL: Adipose triglycerides lipase, HMGCR: HMG-CoA Reductase enzyme, GLUT4: glucose uptake through the transporter, mTOR: Mammalian target of rapamycin, FOXOs: Forkhead box, PGC-1α: Peroxisome proliferator-activated receptor γ coactivator 1α, NRF2: Nuclear factor erythroid 2–related factor 2, SIRTs: Sirtuins, IGF-1: Insulin–insulin-like growth factor 1, ULK1: is an enzyme that in humans is encoded by the ULK1 gene, LKB1: liver kinase B1, ATP: Adenosine triphosphate, CR: Calorie restriction.
